# Contribution of inflammation markers and quantitative sensory testing (QST) indices of central sensitisation to rheumatoid arthritis pain

**DOI:** 10.1186/s13075-024-03407-5

**Published:** 2024-10-08

**Authors:** Vasileios Georgopoulos, Stephanie Smith, Daniel F. McWilliams, Eamonn Ferguson, Richard Wakefield, Dorothy Platts, Susanne Ledbury, Deborah Wilson, David A. Walsh

**Affiliations:** 1 Division of Rheumatology, Orthopaedics and Dermatology, School of Medicine, University of Nottingham, Nottingham, UK; 2 Pain Centre Versus Arthritis, University of Nottingham, Nottingham, UK; 3 NIHR Nottingham Biomedical Research Centre, University of Nottingham, Nottingham, UK; 4 School of Psychology, University of Nottingham, Nottingham, UK; 5 Leeds Institute of Rheumatic and Musculoskeletal Medicine and NIHR Leeds Biomedical Research Centre, University of Leeds, Leeds, UK; 6 Independent Consultant, Nottingham, UK; 7 Rheumatology, Sherwood Forest Hospitals NHS Foundation Trust, Sutton-in-Ashfield, UK; 8 Clinical Sciences Building, A25 Academic Rheumatology, City Hospital, Nottingham, NG51PB UK

**Keywords:** Rheumatoid arthritis, Disease activity, Inflammation, Central sensitisation, Quantitative sensory testing, Pain

## Abstract

**Background:**

Pain, the primary complaint in rheumatoid arthritis (RA), is multifaceted, and may be driven by inflammatory disease activity and central sensitisation. We aimed to ascertain what proportion of RA pain severity is explained by markers of inflammation and quantitative sensory testing (QST) indices of central sensitisation.

**Methods:**

This was a cross-sectional analysis of data from individuals with clinically active RA. Pain severity was assessed using numerical rating scales and inflammation via 28-joint Disease Activity Score (DAS28) and Ultrasound (Greyscale, Power Doppler). Pain sensitivity was assessed by ‘static’ (tibialis anterior or brachioradialis pressure pain detection threshold-PPT-TA/PPT-BR) and ‘dynamic’ (temporal summation-TS, conditioned pain modulation-CPM) QST. Bivariate associations used Spearman’s correlation coefficients, and multivariable linear regression models determined relative contributions to pain severity.

**Results:**

In bivariate analyses of *N* = 96 (age 65 ± 10y, 77% females) people with RA, pain severity was significantly associated with inflammation indices (*r* = 0.20 to 0.55), and CPM (*r*=-0.26). In multivariable models that included TS, CPM, age, sex, and body mass index, inflammation indices remained significantly associated with pain severity. Multivariable models explained 22 to 27% of pain variance. Heterogeneity was apparent for associations with pain between subscores for pain now, strongest or average over the past 4-weeks.

**Conclusions:**

In individuals with clinically active RA, markers of inflammatory disease activity best explain RA pain with only marginal contributions from QST indices of central sensitisation. Although inflammation plays a key role in the experience of RA pain, the greater proportion of pain severity remains unexplained by DAS28 and ultrasound indices of inflammation.

**Supplementary Information:**

The online version contains supplementary material available at 10.1186/s13075-024-03407-5.

## Background

Rheumatoid arthritis (RA) is the commonest inflammatory joint disease, and has major impact on individuals and health services [1]. RA is characterised by inflammation of the synovial joints, swelling, and increased risk of joint damage. Pain is a predominant symptom in people with RA, significantly impacting their quality of life and functional ability. Despite adequate control of inflammation, persistent RA pain indicates contributions from multiple mechanisms, and is mediated by a complex interplay of neurobiological processes within the peripheral and central nervous system, as well as by immunological and psychosocial factors [2].

In rheumatoid synovitis, the release of inflammatory mediators, such as cytokines, prostaglandins and bradykinin, may sensitise nociceptors and amplify pain signals from the joints to the spinal cord, where enhanced excitability of spinal and supra-spinal neurons may lead to a disproportionately severe and widespread pain [3]. Despite the successful mapping of such mechanisms, the exact pathophysiology of RA pain is not entirely understood. Pain in RA can fluctuate from day to day and is characterised by flares, which are not always associated with noticeable joint swelling or an increase in inflammation markers in the blood [4]. This suggests the contribution of diverse underlying mechanisms to the overall pain experience in clinically active RA.

Combining multiple discrete measures provides the most comprehensive evaluation of disease activity in people with RA [5]. The 28-joint disease activity score (DAS28) has been widely used to provide an overall measure of RA disease activity [6]. DAS28 is derived from a non-graded 28-joint count of swollen and tender joints, alongside the erythrocyte sedimentation rate (ESR) or serum C-reactive protein (CRP) concentration, and a general health assessment using a 10 cm Visual Analogue Scale (VAS-GH). Clinically active rheumatoid arthritis can be classified by DAS28 ≥ 2.6 [6].

Ultrasonography can enhance inflammation detection and measurement when compared to clinical examination alone, and is considered another useful tool for monitoring disease activity [7]. However, ultrasound measures of inflammation do not always correlate with other markers of disease activity in people with RA [8].

Quantitative Sensory Testing (QST) is a reliable and valid method to indicate pain sensitivity mediated by the central nervous system (CNS) [9]. Different QST modalities, static or dynamic, assess different aspects of central pain processing [10], and are predictive of pain severity across musculoskeletal conditions [11]. Specifically, in people with RA, QST evidence has indicated that CNS mechanisms of pain sensitivity contribute to RA pain [12].

Joint tenderness and VAS-GH may also be influenced by CNS pain processing, and the difference between 28-joint tender and swollen counts (tender–swollen difference, TSD) has been used to indicate possible contributions from CNS pain processing to clinically assessed disease activity in RA [13].

We hypothesised that inflammation and central pain sensitivity contribute to the pain experience of people with RA. This study aimed to ascertain the contribution of a wide array of inflammatory markers and indices of central sensitisation to pain severity in RA.

## Methods

Study methods and results are reported following the Strengthening the Reporting of Observational Studies in Epidemiology (STROBE) Statement for observational studies [14] and adhere to an a priori registered protocol (Clinicaltrials.gov: NCT04515589).

### Participants and study design

We here present a cross-sectional analysis of baseline data from participants recruited to the Central Aspects of Pain in Rheumatoid Arthritis (CAP-RA) observational study [15]. Participants were adults (≥ 18 years old) with a physician diagnosis of RA and lived experience of pain of > 3/10 on a 0–10 numerical rating scale (NRS). This enabled enrichment of the study population with people with clinically active RA (DAS28 ≥ 2.6), but participants were not excluded if DAS28 < 2.6 was found at study baseline assessment. Participants were recruited within Nottinghamshire, United Kingdom, between August 2021 and August 2023 through secondary care rheumatology services of the Sherwood Forest Hospitals NHS Foundation Trust. After providing informed consent, participants underwent clinical assessment including physical examination for disease activity, a battery of QST modalities, ultrasound imaging and laboratory testing to ascertain current levels of inflammation and were asked to provide information about their pain. Participants were asked to indicate their ethnic origin or background from a fixed set of categories; Asian, Back, White, Other. A free text section was provided for those indicating ‘Other’ as their ethnic origin or background.

### Assessment of pain severity

Pain severity was assessed with three 11-point NRS, which rated `current pain’, `strongest pain over the past 4 weeks’, and `average pain over the past 4 weeks’ respectively, with 0 indicating no pain and 10 the worst pain imaginable [16]. A single `summated pain severity’ score was derived from the total sum (0–30) of these three items as a marker of overall pain severity.

### Assessment of disease activity

Disease activity was assessed using the DAS28 tender (TJC) and swollen (SJC) joint counts. The 10 metacarpophalangeal (MCP), 10 proximal interphalangeal (PIP), 2 wrist, 2 elbow, 2 shoulder and 2 knee joints were examined for tenderness and swelling [17]. Patient Global Assessment of disease activity was also assessed using a 0-100 visual analogue scale (VAS-GH), with 0 indicating best imaginable health state and 100 the worst imaginable health state. Laboratory testing for inflammatory biomarkers was performed in the Department of Pathology, Sherwood Forest Hospitals NHS Foundation Trust, and included ESR derived from whole blood, and CRP using a sandwich ELISA with serum.

Ultrasound imaging was conducted by trained researchers using a modified Backhaus-7 protocol [18] involving palmar and dorsal ultrasound scans of MCP2, MCP3, PIP2, PIP3 joints in both hands and perpendicular scans of the second and fifth metatarsophalangeal (MTP2, MTP5) joints in both feet. To allow comparison with DAS28 tender and swollen joint count, the supra-patellar pouch of the patellofemoral joint, as well as the medial and lateral tibiofemoral joint lines in both knees, were also scanned. All scans were conducted in grayscale and power Doppler modes for the evaluation of synovial hypertrophy (US-SH) and power Doppler (US-PD) in each joint. US-SH and US-PD scoring as well as an overall combined score (US-Comb) was derived for each joint according to EULAR-OMERACT criteria (SH: 0–3, PD: 0–3, Combined: 0–3 for each joint or image) [19]. Twelve-joint tender, swollen, and ultrasound scores (D12) were derived from each of the TJC, SJC, and US-SH and US-PD inflammation grades of these 12 joints (MCP2, MCP3, PIP2, PIP3, wrist and knee, bilaterally).

DAS28 and ultrasound were undertaken by two independent observers (VG, SS) at the same study visit with 25 participants. Intra-class correlation coefficients (ICC) for DAS28-CRP, DAS28-ESR, US-SH, US-PD, and US-Comb showed excellent interrater reliability (DAS28-CRP: 0.92; 95% CI: 0.84 to 0.97, DAS28-ESR: 0.95; 95% CI: 0.90 to 0.98, US-SH: 0.83; 95% CI: 0.69 to 0.94, US-PD: 0.86; 95% CI: 0.68 to 0.94, US-Comb: 0.81; 95% CI: 0.60 to 0.92).

### Assessment of central pain sensitivity

Pain sensitivity was assessed using “static” (Pressure Pain detection Threshold; PPT) and “dynamic” (Temporal Summation; TS, Conditioned Pain Modulation; CPM) QST modalities [10, 20, 21]. QST was undertaken separately by two observers (VG, SS). Participants were requested to have their eyes closed during QST.

#### Pressure pain detection threshold

A handheld digital algometer (Medoc-AlgoMed Advanced Medical Systems – Computerized Pressure Algometer, Israel) featuring a 1 cm-diameter probe was applied, at a constant incremental rate of 50 kPa/sec, at the tibialis anterior muscle (PPT-TA) of the dominant leg, and the brachioradialis muscle in the opposite forearm (PPT-BR), approximately 5 cm distal to the lateral epicondyle. Participants were instructed to activate a hand-held device when the sensation of pressure became painful. PPT was taken as the arithmetic mean of 3 replicate measurements at each testing site. Low PPT indicated greater pain sensitivity.

#### Temporal summation

A single punctate stimulus (256mN) using the retractable blunt needle of a specially manufactured pen (MRC Systems GmbH – The Pin Prick, Germany) was applied on the skin over the patella ligament of the dominant side, followed by 10 repetitive stimuli at a rate of 1/sec. Immediately after the single stimulus, as well as after the 10 repeated stimuli, each participant was asked to rate the experienced intensity of pain or sharpness (single sensation for single stimulus and average of 10 for repeated stimuli respectively) on a paper copy of a 10 cm VAS. TS was calculated as wind-up difference (TS^WUD^ = average of 10 stimuli – single stimulus). The average of the two TS^WUD^ values was used for analysis. Larger positive values of TS indicated greater sensitivity.

#### Conditioned pain modulation

The arithmetic mean of the three replicate PPT measurements (PPT^Mean^ – see above) was used as the unconditioned stimulus. The conditioned PPT (PPT^Con^) was assessed by a single application of the algometer over the tibialis anterior muscle, while contralateral forearm ischemic pain (rated as 4 on an 11-point (0 to 10) current pain NRS) was used as the conditioning stimulus via the application of a 15 cm cuff similar to those used to measure blood pressure, and the simultaneous repeated squeezing of a foam ball [22]. CPM was the difference between PPT^Mean^ and PPT^Con^. A lower positive or more negative CPM value indicated higher sensitivity (less efficient CPM).

ICC between the 2 assessors for 25 participants assessed on the same day, showed fair-to-excellent inter-rater reliability (PPT-TA: 0.85; 95% CI: 0.69 to 0.93, PPT-BR: 0.77; 95% CI: 0.49 to 0.70, TS: 0.50; 95% CI: 0.13 to 0.74, CPM: 0.48; 95% CI: 0.13 to 0.73).

As an additional index of central pain sensitivity in people with RA, we calculated the Tender-Swollen difference (TSD) by subtracting SJC from TJC as measured via DAS28. The TSD has been previously found to predict pain outcomes in people with RA [13].

## Analysis

Presented data are means ± standard deviation (SD) or medians with interquartile range (IQR). Unadjusted associations are presented as Spearman rank-order (ρ) correlation coefficients. Associations were considered little or zero, fair, moderate to good, and good to excellent when ρ values were between 0.00 and 0.25, 0.26 to 0.50, 0.51 to 0.75, and > 0.75, respectively [23].

In regression modelling, the summated pain score was the dependent variable for primary analyses. Independent variables demonstrating an unadjusted correlation with summated pain score at a level of significance of *p* ≤ 0.10 were included in the model [24]. In secondary analyses, separate models were explored for each pain severity subscore as the dependent variable (current pain, strongest pain over the past 4-weeks, average pain over the past 4-weeks). Some components of DAS28 may be measures of pain (VAS-GH, TJC), and markers of disease activity thought to specifically indicate inflammation (CRP, DAS28-SJC, US-SH, and US-PD) were therefore used as independent variables, alongside indices of pain sensitivity (QST, TSD). In sensitivity analyses, models were adjusted for age, sex, and body mass index (BMI). Goodness of model fit and the explanatory power of regression models were evaluated using the coefficient of determination (adjusted R^2^) [24]. Normality testing was conducted with the Shapiro-Wilk test [25], and positively skewed variables found to significantly deviate from normality were transformed. Correlation coefficients and regression coefficients were adjusted after multiple comparisons according to Benjamini and Hochberg [26].

All analyses used R (version 4.3.2) [27] and p-values of ≤ 0.05, after adjusted for multiple comparisons, were taken to indicate statistical significance. Significant correlations or associations are indicated by bold font in tables. Post hoc power calculations were conducted with G*Power software (version 3.1.9.7) [28].

## Results

### Demographic and clinical characteristics

Ninety-two people with RA pain (mean age: 65 ± 10y, 78% female, 100% White) contributed data (Supplementary Fig. 1). Table 1 gives population demographic and clinical characteristics. Mean or median scores indicated moderate pain severity and moderate disease activity. Forty-nine (53%) participants had moderate disease activity (DAS28-CRP > 3.2 to ≤ 5.1), 9 (10%) were in remission (DAS28-CRP < 2.6), 8 (8%) displayed low (DAS28-CRP = 2.6 to ≤ 3.2), and 26 (28%) high disease activity (DAS28-CRP > 5.1). Methotrexate was the commonly used Disease Modifying Antirheumatic Drug (DMARD) and was used by the majority of participants (53 (58%)). Thirty-five (38%) participants were using more than one DMARD at the time of recruitment.

**Table 1:** Participant demographics and clinical characteristics

Characteristic (Units/possible range)	Mean (± SD), Median (IQR) or *n* (%)
**Number of participants**	92
**Age** (years)	65 (± 10)
**BMI** (kg/m^2^)	28.8 (25.4 to 32.1)
**Female**	72 (78%)
**Ethnicity**	
White	92 (100%)
**Self-reported Pain Severity**	
Pain – Summated (0–30)	20 (16 to 23)
Pain – Now (0–10)	5 (4 to 7)
Pain – Strongest [Last 4-weeks] (0–10)	8 (7 to 9)
Pain – Average [Last 4-weeks] (0–10)	7 (5 to 8)
**Inflammation Markers**	
*Bloods*	
Erythrocyte Sedimentation Rate (mm/hr) †	21.0 (6.0 to 32.3)
C-reactive protein (mg/l)	5.0 (2.0 to 9.0)
*Disease Activity Score – 28*	
DAS28 – Erythrocyte Sedimentation Rate (0-9.4) †	4.8 (3.7 to 5.7)
DAS28 – C-reactive protein (0-9.4)	4.5 (3.8 to 5.2)
Tender Joints (0–28)	11.0 (5.0 to 15.0)
Swollen Joints (0–28)	3.0 (1.0 to 6.0)
VAS Global Health (0-100)	50.0 (30.0 to 64.0)
D12 Tender Joints (0–12) ‡	5.0 (3.0 to 8.0)
D12 Swollen Joints (0–12) ‡	2.0 (0.0 to 4.0)
Tender-Swollen Difference ¥	6.0 (2.0 to 11.0)
*Ultrasound*	
Combined Score (0–48)	25.5 (21.0 to 30.0)
Synovial Hypertrophy (0–48)	26.0 (21.0 to 30.0)
Power Doppler (0–48)	8.0 (4.0 to 13.0)
D12 Combined Score (0–36) ‡	19.0 (16.0 to 23.0)
D12 Synovial Hypertrophy (0–36) ‡	19.5 (16.0 to 23.0)
D12 Power Doppler (0–36) ‡	6.5 (3.0 to 11.0)
**Quantitative Sensory Testing**	
Pain Pressure detection Threshold – Tibialis Anterior (kPa)	236.2 (155.7 to 316.9)
Pain Pressure detection Threshold – Brachioradialis (kPa)	147.9 (102.7 to 215.1)
Temporal Summation (0–10)	1.3 (0.4 to 2.5)
Conditioned Pain Modulation (kPa)	63.1 (4.3 to 140.5)
**Disease Modifying Antirheumatic Drugs** ø	**92 (100%)**
Methotrexate	53 (58%)
Sulfasalazine	20 (22%)
Adalimumab	14 (15%)
JAK Inhibitor	8 (9%)
Hydroxychloroquine	6 (7%)
Abatacept	3 (3%)
Etanercept	3 (3%)
Leflunomide	2 (2%)
Rituximab	1 (1%)
Sarilumab	1 (1%)
None	0 (0%)

BMI: Body Mass Index, DAS28: Disease Activity Score – 28 Joints, JAK: Janus Kinase, kPa: kiloPascals, VAS: Visual Analogue Scale

† Calculation is based on *n* = 80 people for whom Erythrocyte Sedimentation Rate was available

‡ D12 refers to the number of joint sites shared between Disease Activity Score – 28 and Ultrasound measurements

¥ Refers to the numerical difference between tender and swollen joint counts

ø *n* = 35/92 (38%) participants were using more than one Disease Modifying Antirheumatic Drug (combination therapy) at the time of recruitment

### Inter-correlation between indices of pain, disease activity or central pain sensitivity

Summated pain severity scores deviated significantly from normality before (W = 0.95, *p* = 0.002), but not after transformation (W = 0.99, *p* = 0.46) (Supplementary Fig. 2). Inter-correlation between pain NRS scores or biomarkers was consistent with their validity as indices of pain, inflammation, disease activity or central pain sensitivity. The 3 NRS scales for pain were inter-correlated, supporting their synthesis to a summated score (possible range 0 to 30) for primary pain analysis (Table 2).

**Table 2:** Correlation matrix for pain sensitivity testing with clinical pain severity

		Pain severity				QST			
		**Summated pain score** (0–30)	**Pain**_**Now**_ (0–10)	**Pain**_**Strongest**_ (0–10)	**Pain**_**Average**_ (0–10)	**PPT**_**Brachioradialis**_ (kPa)	**TS** (0–10)	**CPM** (kPa)	**TSD** (-28 to 28)
		**Cor**	**Cor**	**Cor**	**Cor**	**Cor**	**Cor**	**Cor**	**Cor**
**Clinical Pain Severity**	**Pain**_**Now**_ (0–10)			0.60***	0.66***				
	**Pain**_**Strongest**_ (0–10)				0.65***				
**Pain Sensitivity**	**PPT**_**Tibialis Anterior**_ (kPa)	-0.09	-0.14	-0.11	-0.04	0.76***	-0.27*	0.14	-0.16
	**PPT**_**Brachioradialis**_ (kPa)	-0.10	-0.10	-0.16	-0.04		-0.25*	0.31**	-0.03
	**TS** (0–10)	0.18	0.21*	0.24*	0.09			-0.11	-0.02
	**CPM** (kPa)	-0.29**	-0.22*	-0.30**	-0.29*				0.08
	**TSD** (-28 to 28)	0.04	0.18	-0.04	-0.02				

Cor: Spearman’s Rank Order Correlation, CPM: Conditioned Pain Modulation, kPa: kiloPascals, PPT: Pain Pressure Detection Threshold, QST: Quantitative Sensory Testing, TS: Temporal Summation, TSD: Tender-Swollen Joint Count Difference

All p-values have been corrected for multiple comparisons (Benjamini-Hochberg)

Values in bold indicate statistical significance. * ≤0.05, ** <0.01, ***<0.001

Markers of inflammation and DAS28 components were inter-correlated in the expected direction (Supplementary Table 1). For the 12 joints both with ultrasound scores and TJC/SJC available (D12: MCP2, MCP3, PIP2, PIP3, wrist and knee, bilaterally), higher modified US-Comb (EULAR-OMERACT) score was significantly correlated with higher TJC, but the association with higher SJC did not reach statistical significance (Supplementary Table 2). Different QST indices of central pain sensitivity were also inter-correlated in the expected direction (Table 2). TSD was significantly correlated with TJC but correlations with measurements of pain severity and QST indices of sensitivity did not reach statistical significance (Table 2). Older participants, those with higher BMI, and females displayed higher ESR, higher TJC and TSD, and higher PPT respectively (Supplementary Table 3). In bivariate (unadjusted) analyses, most markers of disease activity were associated with summated pain severity score, as well as with indices of central pain sensitivity (Table 3). Indices of central pain sensitivity were also associated with pain severity in the expected way Table 2.

**Table 3:** Correlation matrix for markers of disease activity with pain severity and pain sensitivity

		Inflammation marker										
		**DAS28-ESR** (0-9.4)	**DAS28-CRP** (0-9.4)	**US-Combined** (0–48)	**ESR** (mm/hr)	**CRP** (mg/l)	**TJC** (0–28)	**SJC** (0–28)	**VAS-GH** (0-100)	**US-SH** (0–48)	**US-PD** (0–48)	
**Clinical** **Pain** **Severity**	**Summated Pain Score** (0–30)	0.43***	0.42***	0.18	0.43***	0.43***	0.14	0.29**	0.55***	0.20*	0.27**	
	Pain _Now_ (0–10)	0.44***	0.48***	0.11	0.33***	0.33***	0.27*	0.31**	0.65***	0.11	0.20	
	Pain _Strongest_ (0–10)	0.42***	0.34**	0.28*	0.49***	0.49***	0.06	0.23*	0.36***	0.17	0.28**	
	Pain _Average_ (0–10)	0.29**	0.29**	0.12	0.35***	0.35***	0.06	0.20*	0.45***	0.26*	0.28**	
**Pain Sensitivity**	**PPT**_**Tibialis Anterior**_ (kPa)	-0.35**	-0.28**	-0.01	-0.22*	-0.18	-0.26*	-0.21*	-0.25*	0.03	-0.08	
	**PPT**_**Brachioradialis**_ (kPa)	-0.31**	-0.21*	0.07	-0.26*	-0.19	-0.18	-0.22*	-0.15	0.12	-0.01	
	**TS** (0–10)	0.07	0.09	0.12	-0.01	0.09	0.02	0.02	0.21*	0.08	0.08	
	**CPM** (kPa)	-0.26*	-0.19	-0.06	-0.19	-0.28*	-0.05	-0.28**	-0.07	0.01	-0.15	
	**TSD (-28 to 28)**	0.47***	0.59***	0.01	-0.10	0.05	0.88***	0.18	0.38***	0.02	-0.05	

Data are Spearman’s correlation coefficients. CRP: C-Reactive Protein, Cor: Pearson or Spearman Correlation, CPM: Conditioned Pain Modulation, DAS28: Disease Activity Score – 28 Joints, ESR: Erythrocyte Sedimentation Rate, kPa: kiloPascals, mm/hr: millimetres per hour, mg/l: milligrams per litre, PPT: Pain Pressure Detection Threshold, SJC: Swollen Joints Count, TJC: Tender Joints Count, TS: Temporal Summation, TSD: Tender-Swollen Joint Count Difference, QST: Quantitative Sensory Testing, US-Combined: Ultrasound – Combined EULAR-OMERACT Score, US-PD: Ultrasound – Power Doppler, US-SH: Ultrasound – Synovial Hypertrophy, VAS-GH: Visual Analogue Scale – Global Health

All p-values have been corrected for multiple comparisons (Benjamini-Hochberg)

Values in bold indicate statistical significance. * ≤0.05, ** <0.01, ***<0.001

### Relative contributions of inflammation and central pain sensitivity to pain severity

The sample size (n = 95) was sufficient for 99% power to explain 25% of the variance (R^2^ ≥ 0.25) in multivariable models. Table 4 presents the multivariable linear regression model showing adjusted associations of pain severity with indices of disease activity (DAS28-CRP, US-Combined), and indices of central pain sensitivity (TS, CPM). Higher DAS28-CRP remained significantly associated with higher summated pain severity score. In secondary analyses that replaced DAS28-CRP with the components CRP and SJC, and replaced combined ultrasound score with its component US-SH, US-PD scores (Table 4), CRP and SJC each was significantly associated with summated pain score (β = 0.42, p < 0.001, and β = 0.21, p = 0.03 respectively). US indices of synovitis were not significantly associated with combined pain severity score in multivariable models that included DAS28-CRP or its CRP and SJC components. Inclusion of age, sex, and BMI did not substantially affect results (Table 4). Each multivariable model explained 22 to 27% of pain variance. Multivariable models for each pain severity subscale displayed similar associations of inflammatory indices (Table 5) and, in addition, TS was consistently associated with `pain now’.

**Table 4:** Multivariable models exploring the relationship between pain severity and markers of disease activity, pain sensitivity, and anthropometric variables

Multivariate models		Pain severity
		Summated pain score
		(0–30)
		β
**Markers of Disease Activity**	**DAS28-CRP** (0-9.4)	0.45***
	**US-Combined** (0–48)	0.10
	*Adjusted R* ^*2*^	0.22***
**Markers of Disease Activity and Pain Sensitivity**	**DAS28-CRP** (0-9.4)	0.44***
	**US-Combined** (0–48)	0.12
	**TS** (0–10)	0.13
	**CPM** (kPa)	-0.14
	*Adjusted R* ^*2*^	0.26***
**Discrete Markers of Inflammation and Pain Sensitivity**	**CRP** (mg/l) †	0.42***
	**SJC** (0–28) †	0.21*
	**US-SH** (0–48) †	0.19
	**US-PD** (0–48) †	-0.08
	**TS** (0–10)	0.13
	**CPM** (kPa)	-0.02
	*Adjusted R* ^*2*^	0.24***
**Markers of Disease Activity Pain Sensitivity and Anthropometric Variables**	**DAS28-CRP** (mg/l)	0.46***
	**US-Combined** (0–48)	0.11
	**TS** (0–10)	0.15
	**CPM** (kPa)	-0.13
	**Age** (y)	0.10
	**Sex** (f)	0.10
	**BMI** (kg/m^2^)	-0.02
	*Adjusted R* ^*2*^	0.26***

β: standardised regression coefficients, BMI: Body-Mass Index, CPM: Conditioned Pain Modulation, CRP: C-Reactive Protein, DAS28: Disease Activity Score – 28 Joints, kPa: kiloPascals, mg/l: milligrams per litre, PPT: Pain Pressure Detection Threshold, SJC: Swollen Joints Count, TJC: Tender Joints Count, TS: Temporal Summation, US-Combined: Ultrasound – Combined EULAR-OMERACT Score, US-PD: Ultrasound – Power Doppler, US-SH: Ultrasound – Synovial Hypertrophy

† Variables are distinct components of DAS28, taken to indicate inflammation without being influenced by pain severity

All p-values have been corrected for multiple comparisons (Benjamini-Hochberg)

Values in bold indicate statistical significance. * ≤0.05, ** <0.01, ***<0.001

**Table 5:** Multivariable models exploring the relationship between each pain severity subscale and markers of disease activity, pain sensitivity, and anthropometric variables

Multivariate models		Pain severity		
		Pain _Now_	Pain _Strongest_	Pain _Average_
		(0–10)	(0–10)	(0–10)
		β	Β	β
**Markers of Inflammation**	**DAS28-CRP** (0-9.4)	0.53***	0.36***	0.31**
	**US-Combined** (0–48)	0.01	0.12	0.16
	*Adjusted R* ^*2*^	0.27***	0.14***	0.12**
**Markers of Inflammation and Pain Sensitivity**	**DAS28-CRP** (0-9.4)	0.53***	0.32**	0.29**
	**US-Combined** (0–48)	0.02	0.13	0.18
	**TS** (0–10)	0.18*	0.17	-0.01
	**CPM** (kPa)	-0.02	-0.18	-0.20*
	*Adjusted R* ^*2*^	0.30***	0.20***	0.15**
**Discrete Markers of Inflammation and Pain Sensitivity**	**CRP** (mg/l) †	0.36**	0.42***	0.32*
	**SJC** (0–28) †	0.28**	0.14	0.10
	**US-SH** (0–48) †	0.12	0.20	0.22
	**US-PD** (0–48) †	-0.08	-0.06	-0.02
	**TS** (0–10)	0.20*	0.16	-0.01
	**CPM** (kPa)	0.09	-0.06	-0.12
	*Adjusted R* ^*2*^	0.19**	0.24***	0.16**
**Markers of Disease Activity**,** Pain Sensitivity**,** and Anthropometric Variables**	**DAS28-CRP** (mg/l)	0.53***	0.32**	0.32**
	**US-Combined** (0–48)	0.01	0.09	0.22*
	**TS** (0–10)	0.19*	0.25*	-0.02
	**CPM** (kPa)	-0.02	-0.19	-0.18
	**Age** (y)	0.03	0.06	0.17
	**Sex** (f)	0.04	0.16	0.10
	**BMI** (kg/m^2^)	-0.01	-0.01	-0.01
	*Adjusted R* ^*2*^	0.28***	0.22***	0.17**

β: standardised regression coefficients, BMI: Body-Mass Index, CPM: Conditioned Pain Modulation, CRP: C-Reactive Protein, DAS28: Disease Activity Score – 28 Joints, kPa: kiloPascals, mg/l: milligrams per litre, PPT: Pain Pressure Detection Threshold, SJC: Swollen Joints Count, TJC: Tender Joints Count, TS: Temporal Summation, US-Combined: Ultrasound – Combined EULAR-OMERACT Score, US-PD: Ultrasound – Power Doppler, US-SH: Ultrasound – Synovial Hypertrophy

† Variables are distinct components of DAS28, taken to indicate inflammation without being influenced by pain severity

All p-values have been corrected for multiple comparisons (Benjamini-Hochberg)

Values in bold indicate statistical significance. * ≤0.05, ** <0.01, ***<0.001

## Discussion

In this study, we demonstrate that several markers of inflammation and central pain hypersensitivity were associated with pain severity in people with RA. Inflammation markers explained the greatest proportion of summated scores for pain severity, whereas indices of central pain hypersensitivity might explain specific pain characteristics. Together, inflammation markers and indices of central pain hypersensitivity explained approximately 25% of RA pain.

Our data support the view that inflammation is predominant amongst the known drivers for pain in RA. Inflammation may cause RA pain by the generation of mediators within the synovium which activate or sensitise nerves. Specific inhibitors of cytokines and inflammatory cells can reduce RA pain [3, 29]. Our data confirm previous reports [30] that markers of inflammation (SJC, ESR, CRP) are correlated with pain severity, highlighting the role of inflammation in the experience of RA pain. We found that synovial hypertrophy or power Doppler ultrasound scores of inflammatory disease activity were also each associated with pain severity, although the association between combined US score and summated pain severity did not reach statistical significance. Previous studies have shown significant associations between ultrasound and disease activity scores that included inflammation and pain components [31], but did not show significant association between ultrasound scores and self-reported pain [32]. Our findings highlight the complexity of both inflammation and pain, and further research should explore which discrete components of inflammation might contribute to specific aspects of pain.

The association between pain and clinical scores for disease activity such as DAS28 [33] may, in part, also be explained by the inclusion in DAS28 of components that directly assess pain (TJC and VAS-GH), even in the absence of inflammation [34, 35]. Associations between QST and global measures of disease activity demonstrated in previous studies [36, 37] might be explained by non-inflammatory components of disease activity assessment. We similarly found that DAS28-ESR was associated with QST measures of pain sensitivity (PPT and CPM). Furthermore, the more specific markers of inflammation (SJC, ESR or CRP) were also associated with PPT and CPM. QST evidence of pain sensitivity, even at sites remote from affected joints, might therefore, in part, be dependent on inflammatory disease activity, which could affect the contribution of pain sensitivity to the overall pain experience in RA. Systemic inflammation might lead to central pain sensitivity, either directly through the actions of circulating inflammatory mediators [38], or by consequence of persistent nociceptive inputs from chronically inflamed joints [39].

CPM may reflect the efficiency of descending analgesic pathways from the brainstem to the spinal cord, and might also be affected by variation in descending facilitatory modulation [40]. In the current study, less efficient CPM was associated with more severe pain, as measured by the summated pain score and also by each component score. Descending modulation of nociceptive transmission might therefore be implicated across diverse aspects of RA pain. Less efficient CPM was also associated with markers of inflammation. In multivariable models that included both CPM and markers of inflammation, CPM effects on pain lost statistical significance, suggesting collinearity or possible mediation effects. Systemic inflammation or persistent nociceptive drive from inflamed joints might blunt descending analgesia, and therefore contribute to RA pain [3].

TS may reflect sensitisation of nociceptive pathways within the spinal cord [41]. Higher TS was associated with `pain now’, and `strongest pain during the past 4 weeks’, both in bivariate and multivariable models. The contributions of spinal sensitisation to RA pain, therefore, might be not entirely explained by concurrent inflammation. TS was not, however, significantly associated with summated pain score, nor `average pain during the past 4 weeks’, suggesting that its contributions might be restricted to specific aspects of RA pain.

Previous studies have found associations between lower PPT (greater sensitivity) at joints affected by RA with RA pain [42, 43], in part reflecting peripheral sensitisation associated with inflammation. Reduced PPT at sites distant to affected joints might reflect central sensitisation, but might alternatively indicate widespread peripheral pain sensitivity, for example due to genetic constitution, or circulating factors that can sensitise peripheral nociceptors [3, 29, 39]. Pain has previously been associated with lower PPT at sites distant to affected RA joints [36]. In the current study, PPTs at 2 non-articular sites (brachioradialis and tibialis anterior) were not significantly associated with summated pain scores, nor with any of the 3 pain subscores. However, significant associations between PPT and TS or CPM might also indicate that previously observed associations between PPT and pain could be explained by other QST modalities. Alterations in descending pain modulatory pathways and mechanisms distinct from inflammation may best be identified by ‘dynamic’ QST modalities, such as CPM and TS [41].

Close relationships between inflammatory and central pain mechanisms might explain why TSD, calculated from DAS28 components, did not importantly contribute to explaining pain in people with RA in this study, and significant but small contributions from QST indices supports their further refinement as indices of central pain sensitivity in RA. Future studies could explore whether other QST modalities, for example those utilising thermal stimuli, may be more sensitive in identifying the contribution of central pain sensitivity in the overall experience of pain in RA.

Overall, our findings indicate that inflammation is driving a considerable part of RA pain with lesser contribution from central pain sensitivity. They also highlight that a large proportion of pain (≤ 75%) remains unexplained by the markers of inflammation or QST modalities that we applied. Inflammation is complex, and there might be specific molecular inflammatory mediators (e.g., cytokines, growth factors, biolipids) that contribute to RA pain [2]. These might differ from those that drive joint swelling, CRP, synovial hypertrophy or synovial blood flow. Furthermore, psychosocial factors [33] and pharmacological or non-pharmacological analgesic strategies [44] that were not adequately captured in our cohort, might also directly modulate the RA pain experience.

Our study has some strengths but is also subject to several limitations. Although our study was designed with adequate power to include a range of established measures of inflammation and central pain hypersensitivity, a larger study including other variables such as, negative affect (depression, anxiety), maladaptive beliefs (catastrophizing), life-style factors (physical activity, sleep quality, smoking status), genetic profiling (or epigenetic profiling), and other pain quality measures, might provide a more complete biopsychosocial profile of individuals with RA, and therefore a more ‘holistic’ view of their pain experience. Our study did not explore analgesic use and how it could moderate the measurements of pain sensitivity and pain severity in our cohort. Future studies should consider investigating actual analgesic consumption rather than prescription and have sufficient numbers for power for individual analgesic classes. We report here analysis of cross-sectional data which maximises participant numbers and study power. Despite this strength, our sample size is too small to adequately explore contribution of inflammation and central pain hypersensitivity in the pain experience of different subgroups (e.g., based on disease activity levels). Our protocol focused on people who might be classified clinically as having active disease and therefore should not be generalised to people with post-inflammatory pain after achieving complete disease remission. Also, exploration of the longitudinal relationships with pain severity may enable greater causal inference about mechanisms driving persistence or resolution of RA pain. For the above reasons, our analyses should be viewed as exploratory, requiring confirmation in a larger independent sample and between multiple time-points.

## Conclusions

In conclusion, inflammation appears to be a strong driver of RA pain, while central pain sensitivity also plays a role, possibly influenced by inflammation’s effects on the CNS and other factors unexplored in the present study. Clinical tools like SJC or CRP, and research tools such as CPM and TS, might help identify these contributions. Recognising the varying levels of inflammation or central pain sensitivity can inform treatment decisions and clinical trial selection. Our findings should help clinicians and patients to understand the complex interplay of pain, inflammation, and central pain sensitivity in people with clinically active RA.

## Electronic supplementary material

Below is the link to the electronic supplementary material.

Supplementary Material 1

## Data Availability

The datasets generated and/or analysed during the current study are not publicly available due to copyright reasons but are available from Professor David Walsh, david.walsh@nottingham.ac.uk on reasonable request.

## Abbreviations

BMI: Body Mass Index
CNS: Central Nervous System
CPM: Conditioned Pain Modulation
CRP: C-reactive protein
DAS28: 28-joint Disease Activity Score
DMARD: Disease Modifying Antirheumatic Drug
ESR: Erythrocyte Sedimentation Rate
ICC: Intra-class Correlation Coefficients
IQR: Interquartile Range
kPa: kiloPascals
MCP: Metacarpophalangeal joints
MTP: Metatarsophalangeal joints
NRS: Numerical Rating Scale
PIP: Proximal Interphalangeal joints
PPT-BR: Pressure Pain detection Threshold - Brachioradialis
PPT-TA: Pressure Pain detection Threshold - Tibialis Anterior
QST: Quantitative Sensory Testing
RA: Rheumatoid Arthritis
SD: Standard Deviation
SJC: Swollen Joint Count
STROBE: Strengthening the Reporting of Observational Studies in Epidemiology
TJC: Tender Joint Count
TS: Temporal Summation
TS^WUD^: Temporal Summation – Wind-up Difference
US-Comb: EULAR-OMERACT overall combined score
US-PD: Ultrasound Power Doppler
US-SH: Ultrasound Synovial Hypertrophy
VAS-GH: Visual Analogue Scale - General Health
