# Supplementary material for: Contribution of inflammation markers and quantitative sensory testing (QST) indices of central sensitisation to rheumatoid arthritis pain
Source: Arthritis Res Ther. 2024 Oct 8;26:175. doi: 10.1186/s13075-024-03407-5 (PMC11460083; doi:10.1186/s13075-024-03407-5)
Supplement: Supplementary file 1 — Supplementary Material 1 [file 13075_2024_3407_MOESM1_ESM.docx]

# Supplementary material


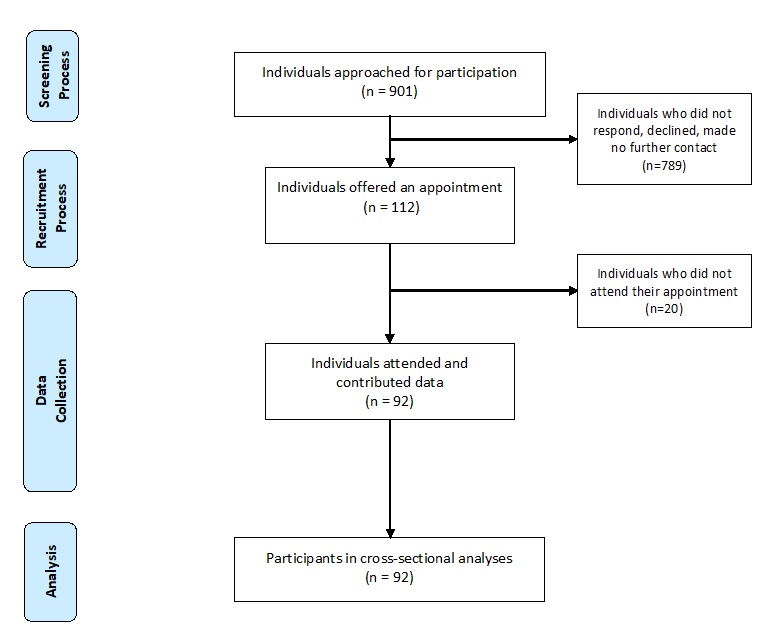


**Supplementary Figure 1. Flow diagram of the eligibility screening, recruitment and data collection processes**

**
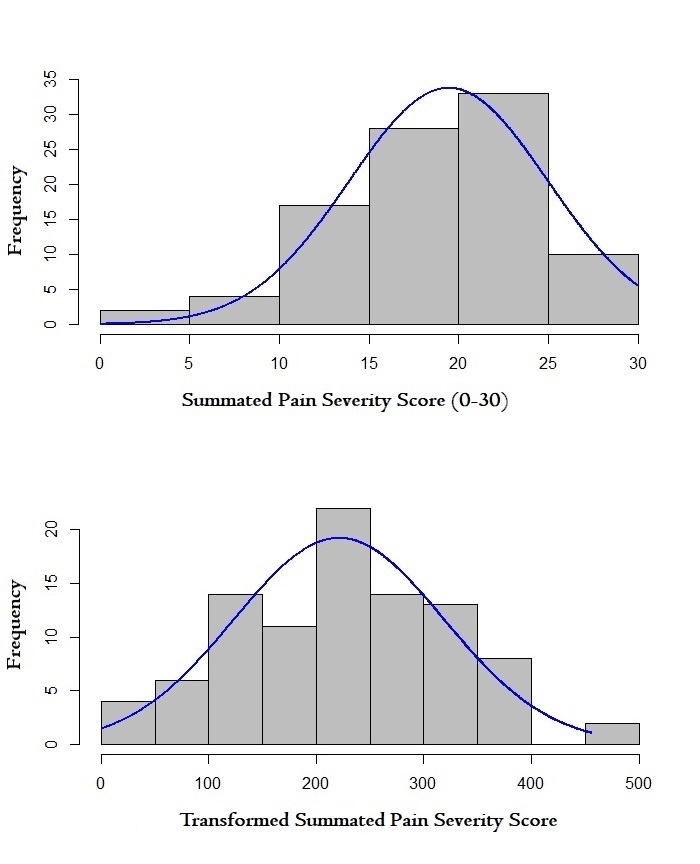
**

**Supplementary Figure 2. Histograms depicting the distribution of the summated pain severity score before and after log-transformation**

**Supplementary Table 1. Correlation matrix for inflammatory markers at baseline.**

|  | | **Inflammatory Markers at baseline** | | | | | | | |
| --- | --- | --- | --- | --- | --- | --- | --- | --- | --- |
|  |  | **ESR**  (mm/hr) | **CRP**  (mg/l) | **TJC**  (0-28) | **SJC**  (0-28) | **VAS-GH** (0-100) | **US-SH**  (0-48) | **US-PD**  (0-48) | **US-Comb** (0-48) |
|  |  | **Cor** | **Cor** | **Cor** | **Cor** | **Cor** | **Cor** | **Cor** | **Cor** |
| **Inflammatory Markers** | **ESR** (mm/hr) |  |  |  |  |  |  |  |  |
|  | **CRP** (mg/l) | **0.64***** |  |  |  |  |  |  |  |
|  | **TJC** (0-28) | 0.01 | 0.10 |  |  |  |  |  |  |
|  | **SJC** (0-28) | 0.15 | 0.12 | **0.59***** |  |  |  |  |  |
|  | **VAS-GH** (0-100) | 0.07 | **0.25*** | **0.44***** | **0.31**** |  |  |  |  |
|  | **US-SH** (0-48) | 0.03 | 0.13 | 0.07 | 0.08 | -0.01 |  |  |  |
|  | **US-PD** (0-48) | **0.38***** | **0.50***** | 0.02 | 0.08 | 0.07 | **0.50***** |  |  |
|  | **US-Comb** (0-48) | 0.04 | 0.18 | 0.06 | 0.08 | 0.06 | **0.92***** | **0.50***** |  |

**CRP:** C-Reactive Protein, **Cor:** Pearson or Spearman Correlation, **ESR:** Erythrocyte Sedimentation Rate, **mm/hr:** millimetres per hour, **mg/l:** milligrams per litre, **SJC**: Swollen Joints Count, **TJC**: Tender Joints Count, **US-SH:** Ultrasound – Synovial Hypertrophy, **US-PD:** Ultrasound – Power Doppler, **US-SH:** Ultrasound – Combined EULAR Score, **VAS-GH**: Visual Analogue Scale – Global Health

All p-values have been corrected for multiple comparisons (Benjamini-Hochberg).

Values in **bold** indicate statistical significance. * ≤0.05, ** <0.01, ***<0.001

**Supplementary Table 2. Correlation matrix for inflammation indices in 12 joints assessed both by ultrasound and by clinical examination.**

|  | | | | **Ultrasound** | | | | | |
| --- | --- | --- | --- | --- | --- | --- | --- | --- | --- |
|  |  |  |  | **Combined**  **(EULAR-OMERACT)** | | **Synovial Hypertrophy** | | **Power Doppler** | |
|  |  |  |  | **Cor** | **p-value** | **Cor** | **p-value** | **Cor** | **p-value** |
| **DAS28** | **Tender Joints** |  | **Total D12^†^ Tender** | **0.21** | **0.04** | 0.18 | 0.08 | 0.04 | 0.69 |
|  |  | **Right** | MCP2 | -0.08 | 0.45 | -0.09 | 0.43 | -0.11 | 0.31 |
|  |  |  | MCP3 | 0.01 | 0.96 | 0.03 | 0.76 | 0.16 | 0.15 |
|  |  |  | PIP2 | 0.16 | 0.14 | 0.13 | 0.23 | 0.16 | 0.12 |
|  |  |  | PIP3 | 0.18 | 0.09 | 0.15 | 0.17 | 0.14 | 0.20 |
|  |  |  | Wrist | 0.12 | 0.28 | 0.12 | 0.28 | 0.05 | 0.65 |
|  |  |  | Knee | -0.07 | 0.55 | 0.17 | 0.12 | -0.10 | 0.40 |
|  |  | **Left** | MCP2 | 0.04 | 0.70 | 0.06 | 0.60 | 0.18 | 0.09 |
|  |  |  | MCP3 | -0.04 | 0.75 | -0.08 | 0.46 | 0.09 | 0.42 |
|  |  |  | PIP2 | -0.02 | 0.84 | -0.03 | 0.81 | -0.06 | 0.59 |
|  |  |  | PIP3 | 0.03 | 0.77 | 0.14 | 0.20 | **0.26** | **0.01** |
|  |  |  | Wrist | 0.11 | 0.31 | 0.07 | 0.50 | 0.17 | 0.12 |
|  |  |  | Knee | **-0.23** | **0.04** | **-0.27** | **0.02** | -0.03 | 0.79 |
|  | **Swollen Joints** |  | **Total D12^†^ Swollen** | 0.16 | 0.12 | 0.16 | 0.14 | 0.13 | 0.23 |
|  |  | **Right** | MCP2 | -0.01 | 0.92 | 0.03 | 0.82 | -0.08 | 0.44 |
|  |  |  | MCP3 | 0.13 | 0.23 | 0.13 | 0.23 | **0.24** | **0.03** |
|  |  |  | PIP2 | -0.01 | 0.91 | -0.02 | 0.82 | 0.09 | 0.41 |
|  |  |  | PIP3 | 0.08 | 0.48 | 0.03 | 0.77 | 0.03 | 0.79 |
|  |  |  | Wrist | 0.11 | 0.29 | 0.04 | 0.72 | 0.19 | 0.07 |
|  |  |  | Knee | 0.11 | 0.37 | 0.01 | 0.99 | 0.16 | 0.16 |
|  |  | **Left** | MCP2 | -0.11 | 0.30 | -0.09 | 0.39 | 0.10 | 0.36 |
|  |  |  | MCP3 | 0.10 | 0.37 | 0.09 | 0.41 | 0.12 | 0.26 |
|  |  |  | PIP2 | 0.01 | 0.97 | 0.01 | 0.98 | -0.04 | 0.69 |
|  |  |  | PIP3 | 0.01 | 0.93 | 0.12 | 0.28 | -0.13 | 0.22 |
|  |  |  | Wrist | -0.02 | 0.89 | 0.03 | 0.75 | 0.16 | 0.13 |
|  |  |  | Knee | -0.10 | 0.40 | -0.17 | 0.15 | -0.03 | 0.80 |

**Combined (EULAR-OMERACT):** Highest score between greyscale and power Doppler is taken as a single score (0-3) for each joint or image, **Cor:** Spearman’s Rank Order Correlation, **DAS28:** Disease Activity Score – 28 Joints, **MCP:** Metacarpophalangeal Joint, **PIP:** Proximal inter-phalangeal

**^†^** Total D12: count of the 12 joints assessed both for DAS28 and by ultrasound

All p-values have been corrected for multiple comparisons (Benjamini-Hochberg).

Values in **bold** indicate statistical significance. * ≤0.05, ** <0.01, ***<0.001

**Supplementary Table 3.** **Correlation matrix for demographic and anthropometric variables with markers of disease activity and measures of pain severity and sensitivity**

| **Markers of disease activity, pain severity, and pain sensitivity** | | **Demographic and Anthropometric Variables** | | | | | |
| --- | --- | --- | --- | --- | --- | --- | --- |
|  |  | **Age** (y) | | **Female Sex** | | **BMI** (kg/m^2^) | |
|  |  | **Cor** | **p-value** | **Cor** | **p-value** | **Cor** | **p-value** |
| **Inflammation Markers** | **DAS28-ESR** (index) | -0.04 | 0.73 | -0.10 | 0.39 | 0.20 | 0.08 |
|  | **DAS28-CRP** (index) | -0.14 | 0.19 | -0.04 | 0.74 | 0.10 | 0.35 |
|  | **US-Combined** (0-48) | -0.14 | 0.17 | -0.04 | 0.71 | 0.05 | 0.62 |
|  | **ESR** (mm/hr) **†** | **0.32** | **<0.01** | -0.02 | 0.85 | 0.03 | 0.83 |
|  | **CRP** (mg/l) | 0.16 | 0.12 | 0.06 | 0.60 | 0.08 | 0.44 |
|  | **TJC** (0-28) | -0.18 | 0.09 | -0.14 | 0.21 | **0.21** | **0.05** |
|  | **SJC** (0-28) | -0.08 | 0.43 | -0.10 | 0.36 | 0.02 | 0.85 |
|  | **VAS-GH** (0-100) | -0.04 | 0.70 | 0.07 | 0.49 | 0.17 | 0.11 |
|  | **US-SH** (0-48) | -0.16 | 0.13 | -0.02 | 0.83 | 0.08 | 0.47 |
|  | **US-PD** (0-48) | 0.02 | 0.87 | -0.06 | 0.61 | -0.04 | 0.69 |
| **Pain** | **Pain _Combined_** (0-30) | 0.09 | 0.41 | 0.12 | 0.25 | 0.01 | 0.89 |
|  | **Pain _Now_** (0-10) | 0.01 | 0.97 | 0.05 | 0.63 | 0.01 | 0.95 |
|  | **Pain _Strongest_** (0-10) | 0.13 | 0.24 | 0.17 | 0.11 | 0.01 | 0.95 |
|  | **Pain _Average_** (0-10) | 0.15 | 0.15 | 0.13 | 0.22 | 0.01 | 0.90 |
| **Pain Sensitivity** | **PPT _Tibialis Anterior_** (kPa) | -0.15 | 0.15 | **0.24** | **0.02** | 0.01 | 0.90 |
|  | **PPT _Brachioradialis_** (kPa) | -0.17 | 0.10 | 0.19 | 0.07 | 0.03 | 0.75 |
|  | **TS** (0-10) | 0.06 | 0.60 | 0.01 | 0.96 | -0.17 | 0.10 |
|  | **CPM** (kPa) | -0.02 | 0.85 | 0.17 | 0.11 | 0.16 | 0.13 |
|  | **TSD** (-28 to 28) | -0.14 | 0.17 | -0.04 | 0.70 | **0.23** | **0.03** |

**BMI:** Body Mass Index, **CPM:** Conditioned Pain Modulation, **CRP:** C-Reactive Protein, **Cor:** Spearman’s Rank Order Correlation, **DAS28:** Disease Activity Score – 28 Joints, **ESR:** Erythrocyte Sedimentation Rate, **kg/m^2^:** kilograms per square metre, **kPa:** kiloPascals, **mm/hr:** millimetres per hour, **mg/l:** milligrams per litre, **PPT:** Pain Pressure detection Threshold, **QST:** Quantitative Sensory Testing, **SJC**: Swollen Joints Count,  **TJC**: Tender Joints Counts, **TS:** Temporal Summation, **TSD:** Tender-swollen Difference, **US-Combined:** Ultrasound – Combined EULAR-OMERACT Score, **US-PD:** Ultrasound – Power Doppler, **US-SH:** Ultrasound – Synovial Hypertrophy, **VAS-GH**: Visual Analogue Scale – Global Health

**†** Calculation is based on n=80 people

All p-values have been corrected for multiple comparisons (Benjamini-Hochberg).

Values in **bold** indicate statistical significance (p<0.05)
